# Timing of cranial radiotherapy in HER2-positive breast Cancer brain metastases treated with Pyrotinib: a multicentre real-world cohort study

**DOI:** 10.1016/j.ctro.2026.101310

**Published:** 2026-10-07

**Authors:** Zhengting Chen, Hao Jing, Sheng Huang, Miao Wang, Dan Mao, Lan Li, Shuang Lu, Xinyan Zhou, Dongyu Bi, Ye Zhang, Xuesong Chen, Yuchao Ma, Yaoxiong Xia, Junlin Yi, Yu Hou

**Affiliations:** a Department of Radiation Oncology, The Third Affiliated Hospital of Kunming Medical University, Yunnan Cancer Hospital, Peking University Cancer Hospital Yunnan, Kunming 650118, China; b Department of Radiation Oncology, National Cancer Center/National Clinical Research Center for Cancer/Cancer Hospital, Chinese Academy of Medical Sciences and Peking Union Medical College, Beijing 100021, China; c Yunnan Key Laboratory of Breast Cancer Precision Medicine, The Department of Breast Surgery, Breast Cancer Center of the Third Affiliated Hospital of Kunming Medical University, Yunnan Cancer Hospital, Peking University Cancer Hospital Yunnan, Kunming 650118, China

**Keywords:** HER2-positive breast cancer, Brain metastases, Radiotherapy, Anti-HER2 therapies, Pyrotinib

## Abstract

Background and purpose: Cranial radiotherapy (RT) and pyrotinib both demonstrate efficacy in HER2-positive breast cancer brain metastases (BCBM); however, the optimal sequencing of these modalities remains to be established. We evaluated survival across treatment sequences within a real-world cohort. Materials and methods:This retrospective, two-centre study included 101 patients with HER2-positive BCBM treated with cranial RT plus pyrotinib (June 2018–July 2025). Based on treatment sequence, patients were categorized into: concurrent RT and pyrotinib (Group A, *n* = 41); RT after intracranial progression on pyrotinib (Group B, *n* = 38); and pyrotinib upon progression following RT (Group C, *n* = 22). The primary endpoint was CNS-PFS. Survival outcomes were assessed via Kaplan–Meier estimation and Cox proportional hazards models.Results: At a median follow-up of 43.3 months, 63 patients (62.4%) had died and 70 (69.3%) experienced CNS-PFS events. Median CNS-PFS was 14.8 months (95% CI 10.68–19.92) overall, and 14.3, 15.0, and 21.4 months in groups A, B, and C, respectively (*P* = 0.252); median OS was 36.0 months (95% CI 28.0–39.7) overall, and 33.0, 35.4, and 42.0 months in groups A, B, and C, respectively (*P* = 0.281). Focal cranial RT was independently associated with longer OS (HR 0.56, 95% CI 0.34–0.94; *P* = 0.028). Any-grade adverse events occurred in 100 of 101 patients (99.0%); grade ≥ 3 events occurred in 2.4%, 7.9%, and 4.5% of groups A, B, and C, respectively (all *P* > 0.05), and radiation necrosis occurred in 7 patients (6.9%). An exploratory threshold of RT initiated >3.9 months before pyrotinib (OS 49.9 vs 29.8 months; HR 0.31, 95% CI 0.11–0.91; *P* = 0.025) did not withstand internal validation.Conclusions: Treatment sequence did not significantly affect OS or CNS-PFS. Focal cranial RT was independently associated with longer survival, and toxicity was manageable. The proposed timing threshold is hypothesis-generating and warrants prospective validation.

## Highlights

• Cranial radiotherapy plus pyrotinib showed a manageable safety profile.
• Focal radiotherapy was independent prognostic factor.
• Earlier radiotherapy was associated with longer survival in exploratory analysis.
• Neither overall survival nor CNS-PFS differed significantly by treatment sequence.

## 1 Introduction

Breast cancer (BC) remains one of the most common malignancies in women worldwide. Brain metastases (BM) constitute a devastating complication of BC, particularly in patients with human epidermal growth factor receptor 2 (HER2)-positive disease, and are associated with dismal 5-year survival rates. HER2 overexpression substantially increases the risk of BM development, with epidemiological studies reporting a cumulative incidence as high as 30–55% among patients with HER2-positive BC [1], [2]. Despite the advent of multiple advanced therapies for breast cancer brain metastases (BCBM)—including whole-brain radiotherapy (WBRT), stereotactic radiotherapy (SRT), and tyrosine kinase inhibitors (TKIs) such as neratinib and pyrotinib—this disease continues to represent a major therapeutic challenge [3], [4], [5]. However, the optimal sequencing of radiotherapy and TKIs remains undefined.

Three principal cranial radiotherapy (RT) modalities are currently available for these patients. Whole-brain radiotherapy (WBRT) treats the entire intracranial compartment and is effective for patients with widespread disease; however, it carries a well-established risk of neurocognitive decline. Stereotactic radiosurgery (SRS) delivers a single, highly conformal ablative dose to discrete lesions, whereas fractionated stereotactic radiotherapy (FSRT) delivers ablative doses in a few fractions and is preferred for larger lesions or those abutting eloquent structures. In contemporary practice, focal FSRT is increasingly favoured for suitable candidates with limited intracranial disease, as randomized trials have demonstrated that SRS alone better preserves neurocognitive function than SRS plus WBRT, without compromising overall survival [6], [7], [8]. WBRT remains appropriate for patients with more extensive intracranial involvement or for those ineligible for focal RT. These considerations provide the rationale for examining how cranial RT should be integrated with pyrotinib-based therapy. In patients with HER2-positive breast cancer and BM, selecting effective systemic therapies is critical [5], [9]. TKIs such as pyrotinib offer a distinct advantage due to their ability to penetrate the blood–brain barrier (BBB)—a key limitation of many other agents. Pyrotinib-based regimens have demonstrated efficacy in HER2-positive BM, particularly in the first- or second-line setting, with a manageable toxicity profile [4], [10]. Cranial RT remains the cornerstone of local management for BCBM. Despite compelling evidence that combining pyrotinib with cranial RT improves local control and may extend survival, the optimal integration schedule of radiotherapy within systemic TKI treatment remains a subject of considerable debate [11], [12], [13]. However, the optimal timing of RT relative to systemic therapy—whether delivered concurrently, deferred until intracranial progression on pyrotinib, or initiated before pyrotinib—remains uncertain, with limited evidence from real-world settings, especially in oligometastatic or asymptomatic scenarios.

To address this gap, we conducted a multicentre retrospective cohort study of patients with HER2-positive BCBM treated with pyrotinib-based therapy (Pyro) and cranial RT. The study objectives were: (1) to compare survival outcomes across three treatment sequences (concurrent RT + Pyro; radiotherapy after pyrotinib progression; pyrotinib after radiotherapy progression); (2) to identify independent prognostic factors; (3) to explore the optimal temporal window for radiotherapy relative to pyrotinib initiation; and (4) to characterise the adverse event profile across treatment groups.

## 2 Materials and methods

### 2.1 Study design and patient selection

This was a retrospective, multicentre observational study. Patients with HER2-positive breast cancer brain metastases (BCBM) treated at the Third Affiliated Hospital of Kunming Medical University and the Cancer Hospital, Chinese Academy of Medical Sciences, between June 2018 and July 2025 were included. The study flow is shown in Supplementary Fig. S1.

Collected data encompassed: patient age; Karnofsky Performance Status (KPS); Modified Breast Graded Prognostic Assessment (mB-GPA); clinicopathological and treatment variables (pathological type, initial TNM stage, lymph node status, presence of vascular tumour thrombus, histological grade, hormone receptor expression status, primary tumour surgical approach, and prior systemic therapies); brain metastasis characteristics (location, number, size, and symptoms); extracranial metastasis status; pyrotinib therapy sequence relative to radiotherapy; radiotherapy parameters and treatment-related toxicities.

This retrospective study was approved by the Medical Ethics Committee of the Third Affiliated Hospital of Kunming Medical University (Approval No. KYLX2024–066). The requirement for informed consent was waived owing to the retrospective nature of the analysis.

Key eligibility criteria were as follows:

1. Age 18–80 years;
2. Histologically confirmed HER2-positive advanced breast cancer, defined as either: HER2 immunohistochemistry (IHC) 3+, or HER2 IHC 2+ with HER2 gene amplification confirmed by in situ hybridization;
3. Eastern Cooperative Oncology Group (ECOG) performance status 0–2, with a life expectancy ≥3 months;
4. Brain metastasis via CT or MRI;
5. At least one measurable intracranial lesion meeting Response Evaluation Criteria in Solid Tumors (RECIST) version 1.1;
6. Completion of at least one cycle of pyrotinib-based targeted therapy;
7. Receipt of cranial radiotherapy following brain metastasis diagnosis;
8. Availability of comprehensive clinical data, including baseline characteristics and follow-up records.

### 2.2 Exclusion criteria

1. Concomitant leptomeningeal metastasis;
2. Uncontrolled BM-related symptoms refractory to maximal medical management of intracranial pressure (e.g., dexamethasone, osmotic agents);
3. Additional primary malignancies (concurrent or historical).

### 2.3 Treatment protocols and definitions

#### 2.3.1 Radiotherapy modality and dose-fractionation schedules

Radiotherapy modality and dose-fractionation were determined by a multidisciplinary team and individualized according to intracranial disease burden and patient condition, rather than by a fixed protocol. Key considerations included: number of BM; maximum lesion diameter and aggregate intracranial tumour volume (estimated from lesion number and size); lesion location (e.g., eloquent cortex, brainstem, posterior fossa); presence and severity of neurological symptoms; performance status and modified Breast Graded Prognostic Assessment (mB-GPA); extent of extracranial disease. In general, focal stereotactic treatmentFSRT—was preferred for patients with a limited number of lesions (typically ≤4), maximum diameter < 3 cm, and limited cumulative tumour volume. Hypofractionated radiotherapy (HFRT) was typically applied for larger lesions or tumors adjacent to critical structures, delivered in >10 fractions with lower total doses than fSRT—was also used as a local cranial radiotherapy option. In this study, SRT is used as an umbrella term for both SRS and FSRT.

Whole-brain radiotherapy (WBRT)—delivered with or without a simultaneous integrated boost (SIB) or sequential boost—was reserved for patients with more numerous or extensive intracranial disease (e.g., >4–10 lesions), lesions unsuitable for focal irradiation due to size or location, uncontrolled or symptomatic intracranial disease, or performance status precluding focal treatment. Modality assignment also reflected physician and patient preference as well as institutional resources; consequently, some patients with limited intracranial disease received WBRT. Treatment allocation was not randomized and remains subject to selection bias (Supplementary Table S1).

### 2.4 Simulation and target delineation

Treatment simulation was performed with patients in the supine position, immobilized using a thermoplastic head–neck–shoulder mask. Localization computed tomography (CT) scans were acquired from the skull vertex to the inferior border of C2 with 3-mm slice thickness. For SRT or boost planning, CT images were co-registered with recent contrast-enhanced T1-weighted magnetic resonance imaging (MRI) to guide target delineation.

For focal radiotherapy, the gross tumour volume (GTV) was defined as the contrast-enhancing lesion on fused MRI. No clinical target volume (CTV) margin was added for single-fraction SRS. For FSRT, the CTV equalled the GTV, and the planning target volume (PTV) was generated with a 1–2 mm isotropic expansion. For WBRT, the clinical target volume encompassed the whole brain including the leptomeninges. Standard WBRT regimens were 30–40 Gy in 10–20 fractions, delivered with or without a SIB of 49.5–60 Gy to gross lesions, or followed by sequential boosts of 10–16 Gy in 5–8 fractions. SRT was delivered as 24 or 30 Gy in 3 fractions, or 30–35 Gy in 5 fractions. Moderately HFRT regimens were delivered in more than 10 fractions, with a lower dose per fraction than SRT (Supplementary Table S2).

### 2.5 Systemic therapy and supportive care

Patients received oral pyrotinib (400 mg once daily; Jiangsu Hengrui Pharmaceuticals Ltd.) for a minimum duration of 6 months. The decision to initiate pyrotinib and the specific treatment indication were determined by the multidisciplinary team. Dosage adjustments were made at the discretion of the treating physician, taking into account the patient's previous treatment regimen, clinical condition, and personal preferences.

Corticosteroids and osmotic agents (dehydrating agents) were administered as clinically indicated for intracranial hypertension, in accordance with institutional practice at both centers. However, data on the proportion of patients exposed, starting dose, cumulative dose, and duration of treatment could not be retrieved retrospectively with sufficient reliability across all 101 patients, and were therefore not included as covariates in the survival analyses.

### 2.6 Endpoints and Follow-up

The primary endpoint was central nervous system progression-free survival (CNS-PFS), defined as the time from initiation of the first study-defined treatment for brain metastases (i.e., whichever was started first, cranial radiotherapy or pyrotinib) to intracranial progression (assessed by Response Assessment in Neuro-Oncology Brain Metastases, RANO-BM) or all-cause death, provided that the event occurred after receipt of both pyrotinib and cranial radiotherapy. Secondary endpoints included overall survival (OS), defined as the time from brain metastasis diagnosis to death from any cause or last follow-up; intracranial efficacy, assessed according to the RANO-BM criteria; and safety, evaluated using the Common Terminology Criteria for Adverse Events (CTCAE) version 5.0. Radiation necrosis (RN) was adjudicated by a multistep approach consistent with the RANO framework: a new or enlarging contrast-enhancing lesion within the previously irradiated high-dose volume, correlated with the radiotherapy treatment plan and further characterized—where available—by advanced imaging (dynamic susceptibility contrast or dynamic contrast-enhanced perfusion MRI with relative cerebral blood volume, MR spectroscopy, or amino-acid PET). Patient follow-up was conducted via telephone, WeChat, and medical record review (outpatient/inpatient records) to document tumour progression and survival status. The follow-up cutoff date was February 24, 2026.

### 2.7 Statistical analyses

Statistical analyses were performed using SPSS 27.0 and R. Baseline characteristics were compared using ANOVA (continuous) and chi-square tests (categorical). Survival was analysed by the Kaplan–Meier method with log-rank testing; univariate Cox regression was used to calculate hazard ratios (HRs) and 95% confidence intervals (CIs). The relationship between radiotherapy timing (continuous) and median progression-free survival was visualised using X-tile. All tests were two-sided, with significance set at *P* < 0.05.

Because the radiotherapy-to-pyrotinib interval cutoff was selected to maximize the log-rank statistic, its apparent significance is subject to selection bias. Three prespecified safeguards were implemented. First, a permutation test (1000 replicates) provided a multiplicity-adjusted *P* value by randomly permuting the timing variable against survival data and re-selecting the optimal cutoff in each replicate. Second, cutoff stability was assessed across 2000 bootstrap samples. Third, optimism in the effect estimate was quantified by bootstrap-based shrinkage, with the corrected HR obtained by subtracting mean optimism from the apparent log HR. To avoid over-reliance on a data-driven split, the timing interval was additionally modelled as a continuous variable (HR per 1-month delay) and with restricted cubic splines (4 knots at the 5th, 35th, 65th, and 95th centiles), and sensitivity analyses were conducted using prespecified clinical cutoffs of 0, 3, 6, and 12 months.

## 3 Results

### 3.1 Patient characteristics and treatment details

Between June 2018 and July 2025, 101 patients met the eligibility criteria and were enrolled. Baseline characteristics are summarised in Table 1. Forty-nine patients (48.5%) were hormone receptor (HR)-positive; 67 (66.3%) had extracranial metastases; and 56 (55.4%) presented with neurological symptoms attributable to brain metastases. Sixty-five patients (64.4%) had ≤4 brain metastases, whereas 36 (35.6%) had >4 lesions, including 9 (9.0%) with >10 lesions. By radiotherapy modality, 84.6% of patients treated with SRT had ≤4 lesions, compared with 50.8% of those receiving WBRT (*P* = 0.001). The number of BM was recorded in categorical ranges rather than exact counts; therefore, individual lesion counts and total intracranial tumour volume were not available. Twenty-nine patients (28.7%) presented with brain metastases as the initial manifestation of cancer. Thirty-six patients (35.6%) had received ≥3 prior lines of systemic therapy. Fifty-eight patients (57.4%) received focal radiotherapy(SRT or HFRT), and 43 (42.6%) received WBRT or WBRT with a boost (Table 1).

**Table 1:** **Baseline characteristics of the study cohort (***N* **= 101).**

Characteristic	Patients, N (%)
Age (years) at diagnosis of BCBM	
≤48	52(51.5)
>48	49(48.5)
Pathological type	
Invasive ductal carcinoma	90(89.1)
Other	11(10.9)
Hormone receptor status	
Positive	49(48.5)
Negative	52(51.5)
Node stage	
N0	14(13.9)
N1	28(27.7)
N2	34(33.7)
N3	25(24.8)
Extracranial metastatic	
Yes	67(66.3)
No	34(33.7)
GPA Score	
0–2.5	42(41.6)
3–4	59(58.4)
LVI	
Yes	19(18.8)
No	71(70.3)
Other	11(10.9)
Number of BM	
≤4	65(64.4)
>4	36(35.6)
Size of BM	
<3 cm	84(83.2)
≥3 cm	17(16.8)
BM present at initial diagnosis of BC	
Yes	29(28.7)
No	72(71.3)
Symptoms caused by BM	
Yes	56(55.4)
No	45(44.6)
Location of BM	
Supratentorial	34(33.7)
Infratentorial and Other	67(66.3)
Lines of systemic treatment	
0–2	65(64.4)
≥3	36(35.6)
RT modalities	
Focal (SRT/HFRT)	58(57.4)
WBRT ± boost	43(42.6)
RT technique	
IMRT/VMAT	71(70.3)
TOMO	18(17.8)
CyberKnife/Unity	8(7.9)
Other	4(4.0)
Treatment sequence	
A: Pyro + RT (concurrent)	41(40.6)
B: Pyro → RT	38(37.6)
C: RT → Pyro	22(21.8)

Abbreviation: BCBM, breast cancer brain metastases; GPA, graded prognostic assessment; NA, not available; HFRT, hypofractionated radiotherapy; Pyro, pyrotinib; LVI, Lymphovascular invasion; RT, radiotherapy; SRT, stereotactic radiotherapy; TOMO, tomotherapy; WBRT, whole-brain radiotherapy.

According to the treatment sequence, patients were categorized into three groups: Group A (concurrent, pyrotinib + radiotherapy; *n* = 41), Group B (radiotherapy after pyrotinib progression, pyrotinib → radiotherapy; *n* = 38), and Group C (pyrotinib after radiotherapy progression, radiotherapy → pyrotinib; *n* = 22). Baseline characteristics were generally balanced across the three groups, except for RT technique( Table 2). However, prior brain-directed therapy (≥3 lines) tended to be more frequent in Group B (50%) than in Group A (24%) and Group C (32%; *P* = 0.055), Similarly, a GPA score of 3–4 was more common in Group A (71%) compared with Group B (47%) and Group C (55%; *P* = 0.100).

**Table 2:** Baseline characteristics according to treatment sequence (*N* = 101).

Characteristic	Subgroup	A:Pyro+RT (*n* = 41)	B:Pyro→RT (*n* = 38)	C: RT → Pyro (*n* = 22)	*P* value
Age	≤48 years	22 (53.7%)	22 (57.9%)	8 (36.4%)	0.257
	>48 years	19 (46.3%)	16 (42.1%)	14 (63.6%)	
LVI	Yes	7 (17.1%)	7 (18.4%)	5 (22.7%)	0.858
	No	33 (80.5%)	23 (60.5%)	15 (68.2%)	
	NA	1 (2.4%)	8 (21.1%)	2 (9.1%)	
N stage	N0	7 (17.1%)	6 (15.8%)	1 (4.5%)	0.355
	N1	11 (26.8%)	11 (28.9%)	6 (27.3%)	
	N2	14 (34.1%)	11 (28.9%)	9 (40.9%)	
	N3	9 (22.0%)	10 (26.3%)	6 (27.3%)	
Stage at diagnosis	I-II	15 (36.6%)	10 (26.3%)	5 (22.7%)	0.438
	III-IV	26 (63.4%)	28 (73.7%)	17 (77.3%)	
ER status	Positive	17 (41.5%)	22 (57.9%)	10 (45.5%)	0.327
	Negative	24 (58.5%)	16 (42.1%)	12 (54.5%)	
GPA score	0–2.5	12 (29.3%)	20 (52.6%)	10 (45.5%)	0.100
	3.0–4.0	29 (70.7%)	18 (47.4%)	12 (54.5%)	
BM location	Supratentorial	12 (29.3%)	15 (39.5%)	7 (31.8%)	0.618
	Infratentorial or other	29 (70.7%)	23 (60.5%)	15 (68.2%)	
BM size	<3 cm	33 (80.5%)	32 (84.2%)	19 (86.4%)	0.819
	≥3 cm	8 (19.5%)	6 (15.8%)	3 (13.6%)	
BM number	≤4	28 (68.3%)	22 (57.9%)	15 (68.2%)	0.574
	>4	13 (31.7%)	16 (42.1%)	7 (31.8%)	
BM number (10)	≤10	39 (95.1%)	33 (86.8%)	19 (86.4%)	0.376
	>10	1 (2.4%)	5 (13.2%)	3 (13.6%)	
BM symptom	Yes	24 (58.5%)	21 (55.3%)	11 (50.0%)	0.809
	No	17 (41.5%)	17 (44.7%)	11 (50.0%)	
Prior systemic therapy	0–2 lines	31 (75.6%)	19 (50.0%)	15 (68.2%)	0.055
	≥3 lines	10 (24.4%)	19 (50.0%)	7 (31.8%)	
Extracranial metastases	Yes	23 (56.1%)	27 (71.1%)	17 (77.3%)	0.175
	No	18 (43.9%)	11 (28.9%)	5 (22.7%)	
Brain-first metastases	Yes	14 (34.1%)	8 (21.1%)	7 (31.8%)	0.41
	No	27 (65.9%)	30 (78.9%)	15 (68.2%)	
RT Modality	WBRT± boost	16 (39.0%)	17 (44.7%)	10 (45.5%)	0.836
	Focal RT (SRT/HFRT)	25 (61.0%)	21 (55.3%)	12 (54.5%)	
RT technique	VMAT/IMRT	31 (75.6%)	28 (73.7%)	12 (54.5%)	0.014
	TOMO	9 (22.0%)	6 (15.8%)	3 (13.6%)	
	CyberKnife/Unity	0	4 (10.5%)	4 (18.2%)	
	other	1 (2.4%)	0	3 (13.6%)	

Abbreviation: ER, estrogen receptor; GPA, graded prognostic assessment; NA, not available; LVI, lymphovascular invasion; Pyro, pyrotinib; RT, radiotherapy; SRT, stereotactic radiotherapy; TOMO, tomotherapy; WBRT, whole-brain radiotherapy. HFRT, hypofractionated radiotherapy.

### 3.2 Survival analysis

#### 3.2.1 Overall cohort

At a median follow-up of 43.3 months (range, 4.6–80.2; last follow-up, February 24, 2026), 63 of 101 patients (62.4%) had died, and intracranial progression or death had occurred in 70 (69.3%). The median CNS-PFS was 14.8 months (95% CI, 11.8–21.4), with estimated CNS-PFS rates of 57.9%, 33.7%, and 16.2% at 12, 24, and 36 months, respectively (Fig. 1A). The numbers of patients at risk were 101, 88, 56, 37, 24, 5, 3, and 0 at baseline and at 6, 12, 18, 24, 36, 48, and 60 months, respectively. The median OS for the entire cohort was 36.0 months (95% CI, 28.0–39.7), with survival rates of 88.1%, 64.8%, 49.4%, and 21.8% at 12, 24, 36, and 60 months, respectively. The numbers of patients at risk were 101, 99, 88, 71, 59, 38, 15, and 6 at baseline and at 6, 12, 18, 24, 36, 48, and 60 months, respectively.

**Fig. 1:**
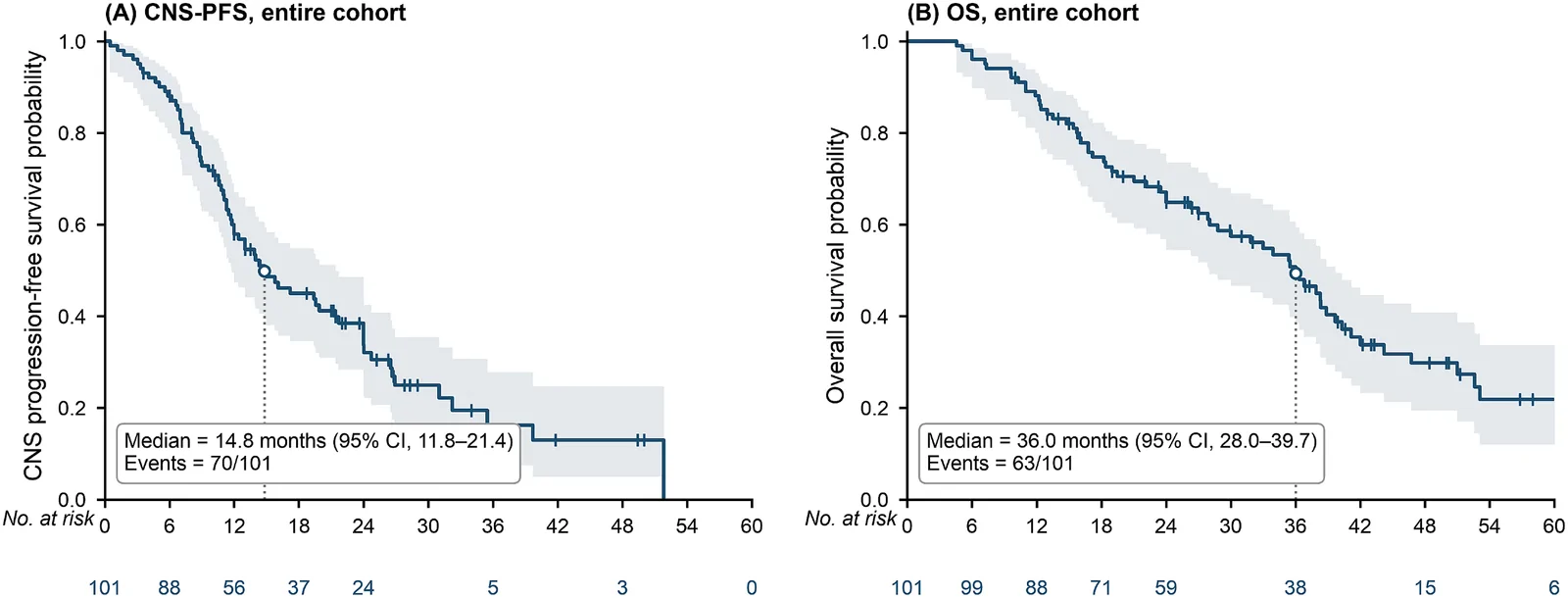
Kaplan–Meier estimates of (A) central nervous system progression-free survival (CNS-PFS) and (B) overall survival (OS) in the entire cohort (*n* = 101). Numbers at risk are shown below each panel.

#### 3.2.2 CNS-PFS by treatment sequence

The median CNS-PFS for the entire cohort was 14.8 months (95% CI, 11.8-21.4.). When stratified by treatment sequence, median CNS-PFS was 14.3 months (95% CI, 11.3–26.6) in Group A (concurrent), 15.0 months (95% CI, 9.0–24.0) in Group B (pyrotinib progression followed by radiotherapy), and 21.4 months (95% CI, 8.9–39.7) in Group C (radiotherapy progression followed by pyrotinib). There was no significant difference in CNS-PFS among the three groups (*P* = 0.252; Fig. 2).

**Fig. 2:**
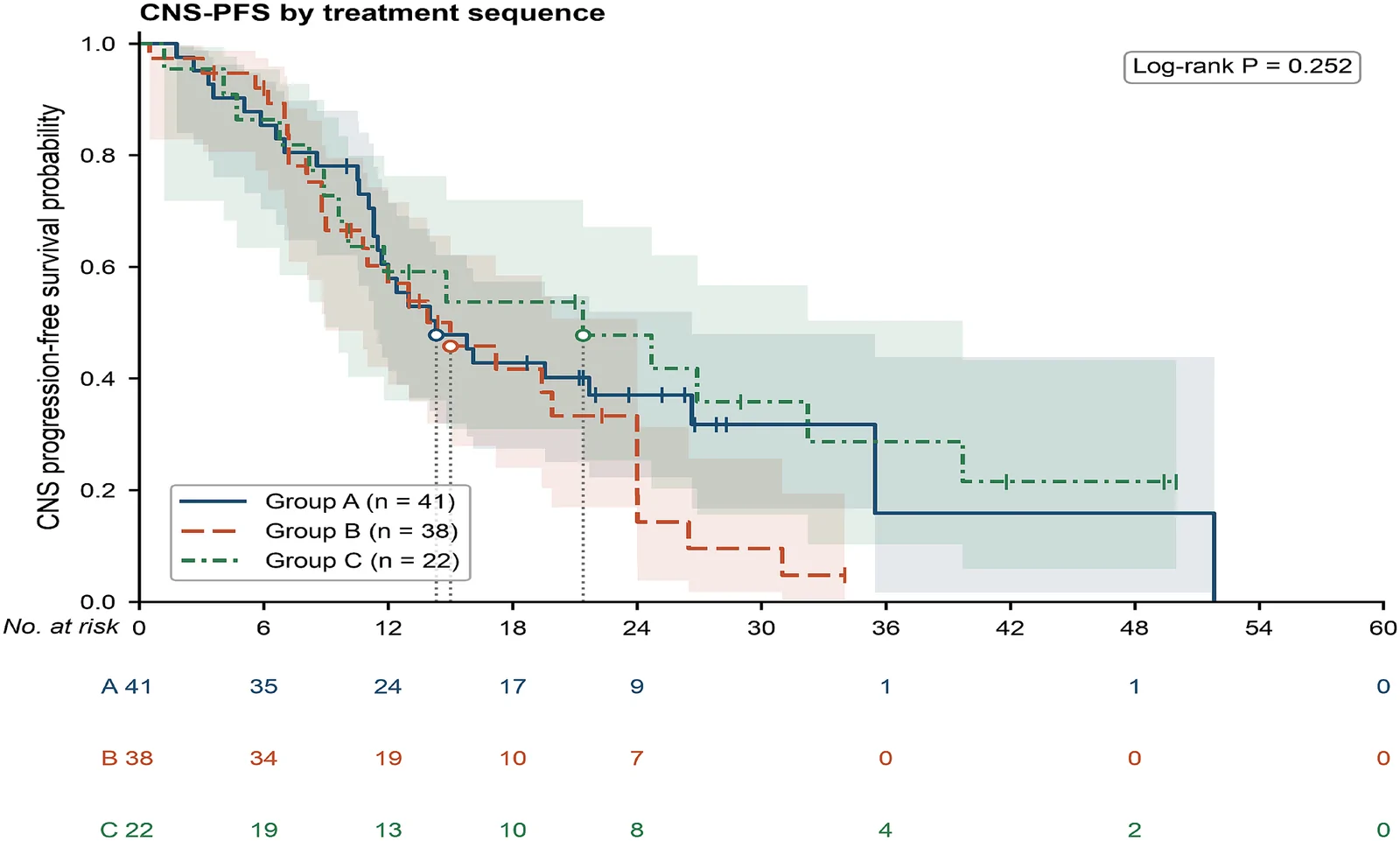
**Central nervous system progression-free survival (CNS-PFS) by treatment sequence.** Group A: concurrent pyrotinib and radiotherapy (Pyro+RT); Group B: radiotherapy after pyrotinib progression (Pyro→RT); Group C: pyrotinib after radiotherapy progression (RT → Pyro). PD, progressive disease; RT, radiotherapy; Pyro, pyrotinib.

### 3.3 Overall survival by treatment sequence

The median OS by treatment sequence group was 33.0 months (95% CI, 20.52–45.48) in Group A (concurrent pyrotinib and radiotherapy), 35.4 months (95% CI, 18.65–45.15) in Group B (pyrotinib followed by radiotherapy), and 42.0 months (95% CI, 28.08–55.92) in Group C (radiotherapy followed by pyrotinib). Although OS appeared longest in Group C, no statistically significant difference was observed among the three groups (*P* = 0.281; Fig. 3).

**Fig. 3:**
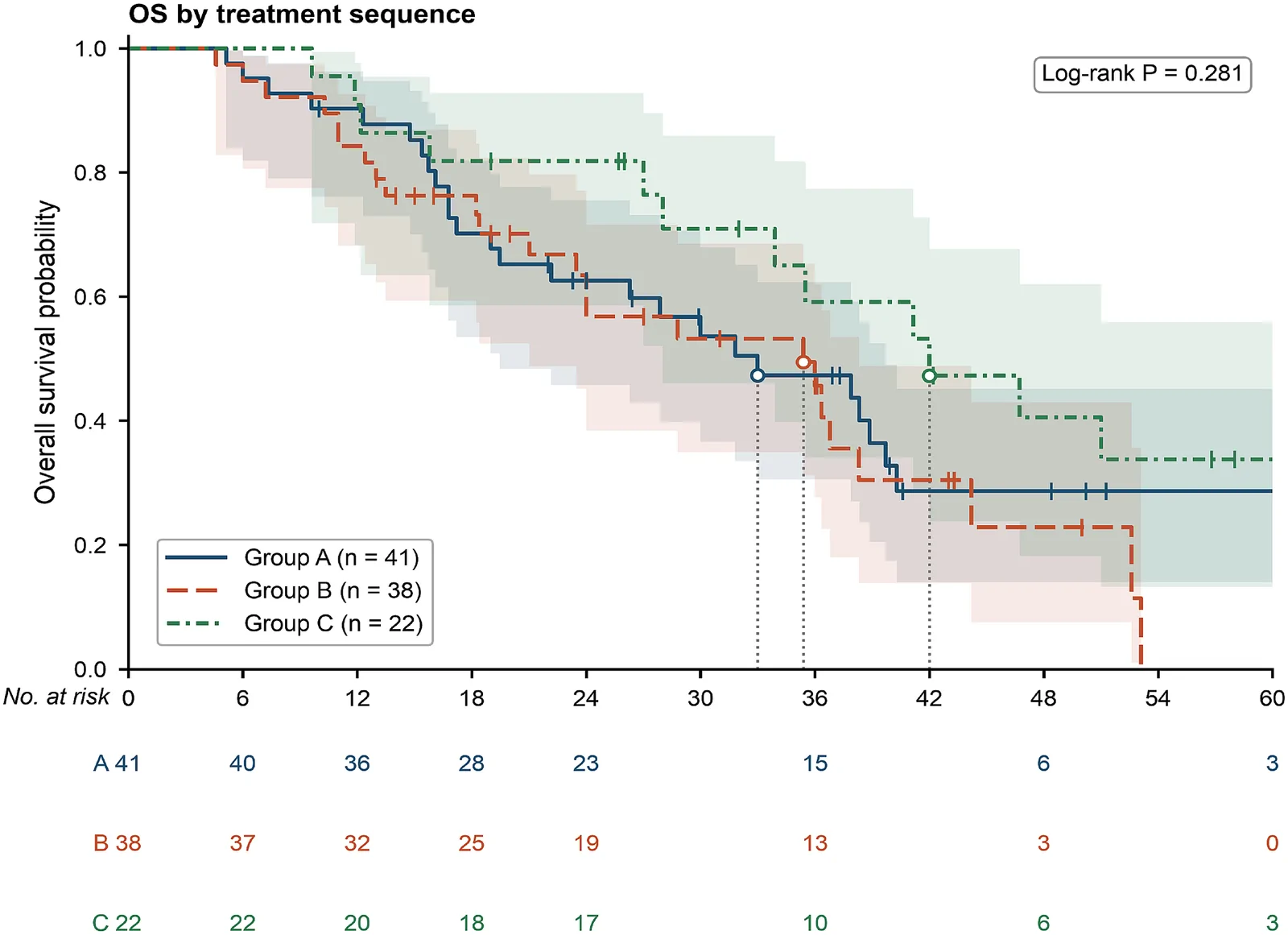
**Overall survival (OS) by treatment sequence**. Group A: concurrent pyrotinib and radiotherapy (Pyro+RT); Group B: radiotherapy after pyrotinib progression (Pyro→RT); Group C: pyrotinib after radiotherapy progression (RT → Pyro). RT, radiotherapy; Pyro, pyrotinib.

### 3.4 Prognostic factors for CNS-PFS and OS

In univariate Cox regression for CNS-PFS, age > 48 years (*P* = 0.094), GPA score 3–4 (*P* = 0.068), and absence of brain metastasis symptoms (*P* = 0.097) showed borderline protective trends (*P* < 0.10).. In multivariate analysis, no variable achieved statistical significance (all *P* > 0.05); Treatment sequence was not significantly associated with CNS-PFS in either univariate (Group B vs. A: HR 1.39, 95% CI 0.81–2.38, *P* = 0.232; Group C vs. A: HR 0.82, 95% CI 0.43–1.56, *P* = 0.543) or multivariate analysis. These findings indicate that the effect of radiotherapy timing on CNS-PFS was overshadowed by stronger prognostic factors—specifically age and radiotherapy modality—after adjustment for baseline characteristics (Fig. 4).

**Fig. 4:**
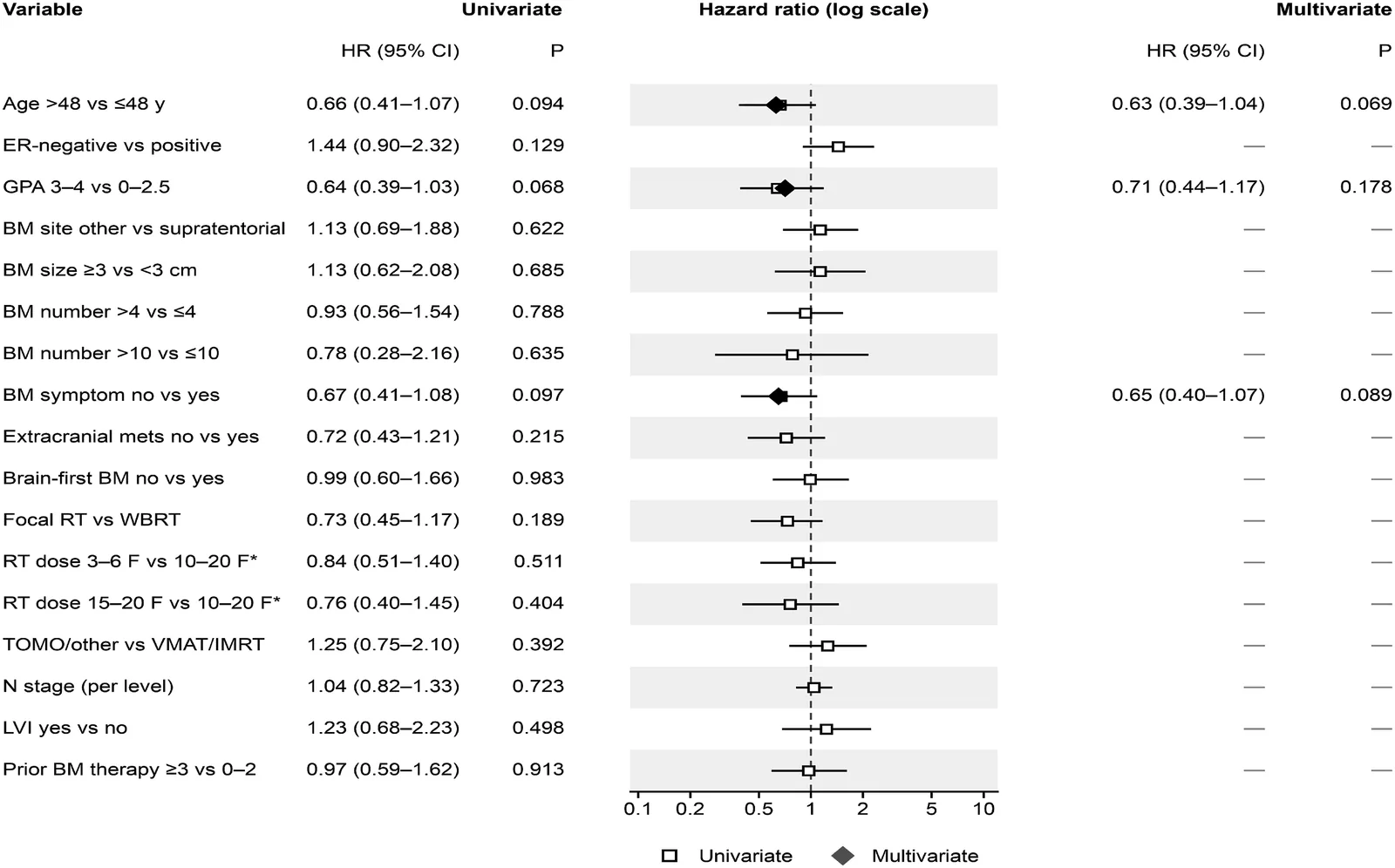
**Forest plots depicting univariate and multivariate analyses of CNS-PFS in BCBM patients treated with cranial radiotherapy and pyrotinib.** No variable was statistically significant (all *P* > 0.05); HRs and 95% CIs are shown. Abbreviation: BCBM, breast cancer brain metastases; GPA, graded prognostic assessment; LVI, Lymphovascular invasion; N, Node; RT, radiotherapy; WBRT, whole-brain radiotherapy.

### 3.5 Prognostic Factors for OS

In multivariable analysis, focal radiotherapy (HR 0.56, 95% CI 0.34–0.94; *P* = 0.028) and age > 48 years (HR 0.56, 95% CI 0.33–0.96; *P* = 0.034) were associated with longer OS (Fig. 5). Because the estimate varied with the adjustment set (HR 0.55–0.61; *P* = 0.023–0.074), we report the full set of models in Supplementary Table S3 and Fig. S2. After propensity score matching the association was similar in magnitude but did not reach conventional significance (HR 0.54, 95% CI 0.29–1.01; *P* = 0.052), whereas it remained significant after inverse-probability-of-treatment weighting (HR 0.52, 95% CI 0.31–0.88; *P* = 0.015). An age-by-GPA interaction (*P* = 0.022) showed that older age benefited only patients with GPA 3–4 (HR 0.32; *P* = 0.003), not GPA 0–2.5 (HR 1.08; *P* = 0.843). This association was stable across adjustment strategies—unadjusted HR 0.55 (95% CI 0.33–0.92; *P* = 0.024); adjusted for GPA and intracranial tumour burden HR 0.54 (95% CI 0.32–0.92; *P* = 0.023); and fully adjusted HR 0.52 (95% CI 0.30–0.91; *P* = 0.021)—which argues against its being explained by measured baseline prognostic factors. These results indicated that the survival advantage associated with older age was confined to patients with GPA 3–4 (HR 0.32, 95% CI 0.15–0.68; *P* = 0.003).

**Fig. 5:**
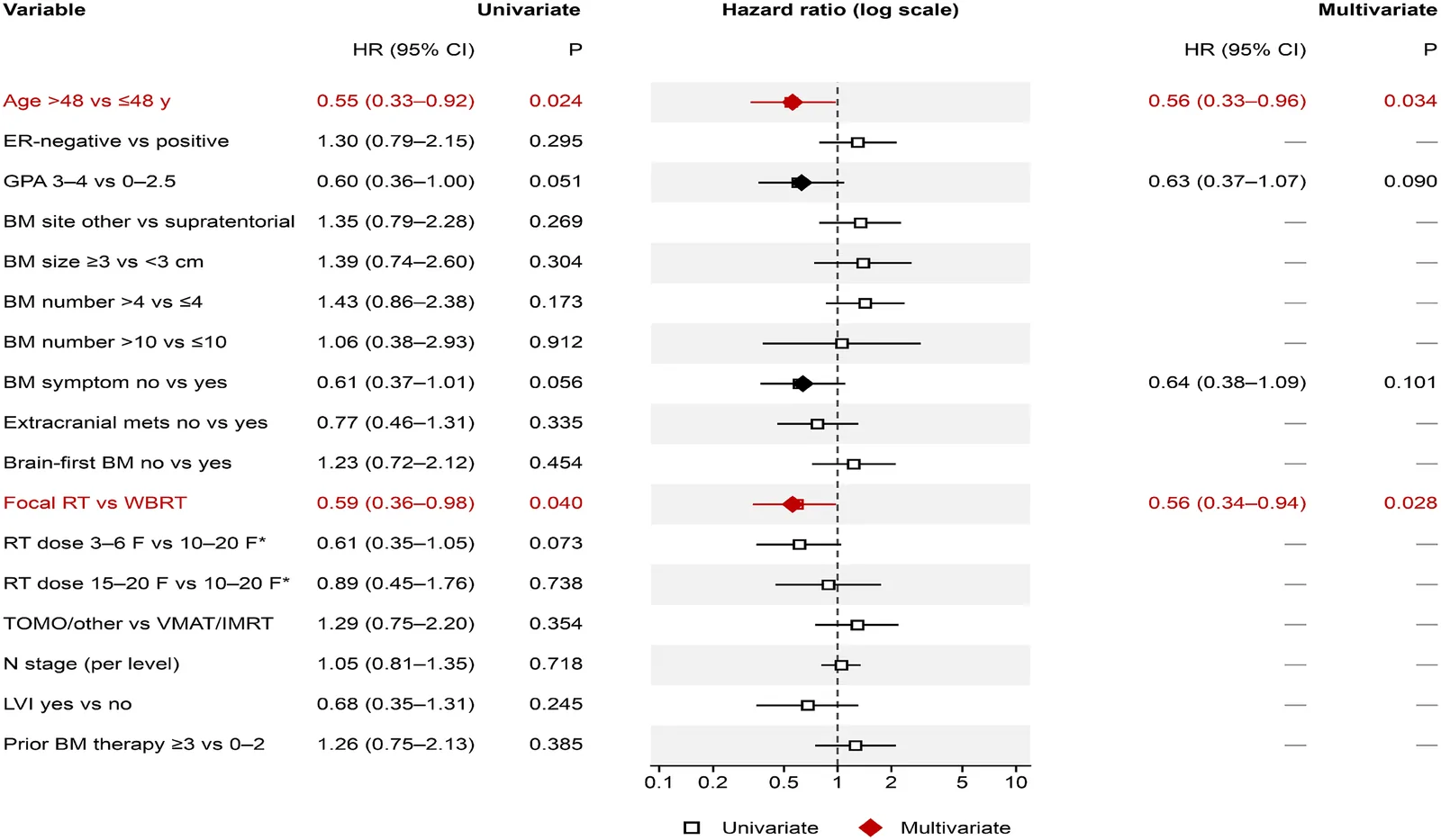
**Forest plots showing univariate and multivariate OS analyses in BCBM patients treated with cranial radiotherapy and pyrotinib**. Abbreviation: BCBM, breast cancer brain metastases; ER, estrogen receptor; N, Node; GPA, graded prognostic assessment; LVI, Lymphovascular invasion; RT, radiotherapy; WBRT, whole-brain radiotherapy.

### 3.6 Optimal cutoff for the timing of radiotherapy relative to pyrotinib

The optimal cutoff for radiotherapy administered prior to pyrotinib was −3.9 months (radiotherapy 3.9 months before pyrotinib), with 13 patients (23.6%) in the favourable window. Median OS was 49.9 vs. 29.8 months (HR 0.31, 95% CI 0.11–0.91; *P* = 0.025). Internal validation demonstrated overfitting: permutation *P* = 0.211; bootstrap optimum highly unstable (median − 4.3 months; IQR −8.7 to 3.1); optimism correction attenuated HR to 0.66. Continuous modelling showed a non-significant trend (per month delay: HR 1.024, 95% CI 0.999–1.051; *P* = 0.065) with non-linearity (*P* = 0.023). Prespecified cutoffs (≥0, ≥3, ≥6, ≥12 months) were directionally consistent but non-significant after Bonferroni correction. The −3.9-month cutoff is strictly hypothesis-generating (Fig. 6; Supplementary Figs. S3.1–2).

**Fig. 6:**
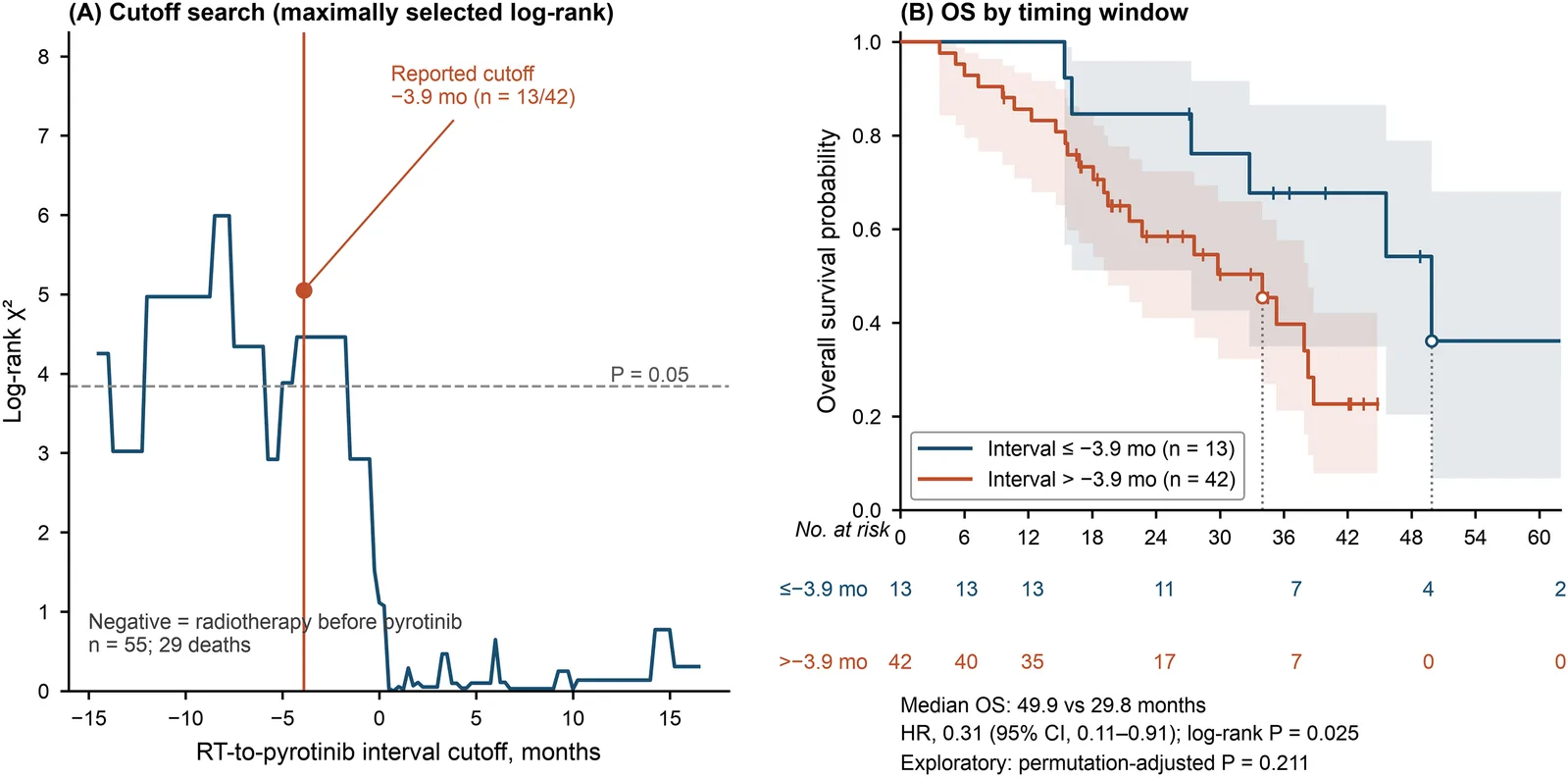
**Timing of radiotherapy relative to pyrotinib.** Optimal cutoff: −3.9 months (radiotherapy before pyrotinib). OS, Overall survival;RT, Radiotherapy.

### 3.7 Safety evaluation

Among the key adverse events (AEs) assessed, no statistically significant differences were observed across the three treatment sequences. AEs were documented in all but one patient (100/101, 99.0%): 41/41 (100.0%), 37/38 (97.4%), and 22/22 (100.0%) in Groups A, B, and C, respectively Table 3. The most common adverse event was gastrointestinal reaction (diarrhea), occurring in 78.0% (32/41) of Group A, 64.9% (24/37) of Group B, and 68.2% (15/22) of Group C; myelosuppression (12.2%–13.6%), neurological reactions (7.3%–16.2%), and fatigue/hand-foot syndrome (2.4%–9.1%) were less frequent. The overall distribution of adverse event types did not differ significantly among the three groups (*P* = 0.767). Five grade 3 events were recorded (1 in Group A, 3 in Group B, and 1 in Group C), corresponding to grade 3 event rates of 2.4%, 7.9%, and 4.5% (A vs B: Fisher exact *P* = 0.347); two of the three grade 3 events in Group B were neurological. No grade ≥ 4 events were observed.

Radiation necrosis (RN), adjudicated as described in the Methods, was documented in 7 patients. By modality, radiation necrosis occurred in 4 of 43 patients (9.3%) after WBRT and 3 of 58 patients (5.2%) after focal radiotherapy (Fisher exact *P* = 0.455). The majority of RN cases were asymptomatic. Treatment was managed stepwise: asymptomatic patients underwent observation with serial imaging; symptomatic perilesional oedema was treated with corticosteroids; and bevacizumab (7.5 mg/kg intravenously every 2–3 weeks for four cycles) was administered for progressive or corticosteroid-refractory necrosis. Surgical resection was reserved for refractory mass effect or when tumour progression could not be excluded clinically. No patient died as a result of RN. Because the date of onset of necrosis was not captured in the database, a competing-risk (Fine-Gray) analysis with death as a competing event could not be performed; given that 6 of the 7 affected patients have died and that necrosis is characteristically a late event, they are likely to underestimate the true cumulative incidence. All treatment-related adverse events were manageable, and no treatment-related deaths occurred (Table 3).

**Table 3:** Adverse events in three groups.

Adverse event(AE)	A: Pyro+RT (*n* = 41)	B: Pyro→RT (*n* = 38)	C: RT → Pyro (*n* = 22)	*P* overall
Adverse events				
Myelosuppression	5 (12.2%)	5 (13.2%)	3 (13.6%)	-
Gastrointestinal (vomiting/diarrhea)	32 (78.0%)	24 (63.2%)	15 (68.2%)	
Neurological (cognition/seizure)	3 (7.3%)	6 (15.8%)	2 (9.1%)	
Fatigue/HFS	1 (2.4%)	2 (5.3%)	2 (9.1%)	
Any AE	41 (100.0%)	37 (97.4%)	22 (100.0%)	0.767
AE grade				
G1	35 (85.4%)	29(76.3%)	17 (77.3%)	0.799
G2	5 (12.2%)	5 (13.2%)	4 (18.2%)	
G3	1 (2.4%)	3 (7.9%)	1 (4.5%)	
Grade ≥ 2	6 (14.6%)	8 (21.1%)	5 (22.7%)	
Grade ≥ 3	1 (2.4%)	3 (7.9%)	1 (4.5%)	
Radiation necrosis	2 (4.9%)	3 (7.9%)	2 (9.1%)	0.786

Abbreviation: AE, Adverse event; G1–3, grade 1–3, HFS, hand-foot syndrome; Pyro, Pyrotinib Therapy; RT, Radiotherapy.

## 4 Discussion

Improved systemic control of extracranial HER2-positive breast cancer has prolonged survival but has simultaneously increased the incidence of BM, whose biology remains incompletely understood [14], [15]. Durable intracranial control therefore requires multimodal strategies that integrate HER2-directed systemic therapy with cranial radiotherapy. In this two-centre cohort of 101 patients with HER2-positive BCBM treated with radiotherapy plus pyrotinib, median intracranial PFS and OS for the whole cohort were 14.8 months and 36.0 months, respectively. Although both endpoints were numerically longest in Group C (pyrotinib initiated after intracranial progression during radiotherapy), the differences among the three treatment-sequence groups did not reach statistical significance. Treatment-related adverse events were manageable and no treatment-related deaths occurred.

The intracranial PFS observed here is consistent with that reported in most series of comparable design, but it is shorter than the 18.0 months of CNS-PFS reported in the phase II BROPTIMA trial. The BROPTIMA trial demonstrated that adding radiotherapy to pyrotinib and capecitabine provided durable intracranial benefit, with a 1-year CNS-PFS rate of 74.9% and a manageable safety profile [11]. The disparity most plausibly reflects differences in case mix rather than in treatment efficacy: the present real-world cohort was enriched in poor-prognosis features, with extracranial metastases present in 66.3% of patients (67/101) and three or more lines of prior systemic therapy in 35.6% (36/101). The prolonged survival achieved with modern systemic therapy nonetheless underscores the need for sustained therapeutic strategies that combine durable efficacy with acceptable toxicity; in this regard, the median PFS of 7.2 months and median OS of 25.2 months reported for pyrotinib rechallenge after progression on prior pyrotinib further attest to the clinical value of continued HER2 blockade in this setting [16].

The survival outcomes observed in the present cohort compare favourably with published benchmarks. The PERMEATE study reported a median OS of 35.9 months and 30.6 months in patients with untreated BM and in those with progression after intracranial radiotherapy, respectively. Against these benchmarks, the overall median OS of 36.0 months and median intracranial PFS of 14.8 months in this cohort exceed the 11.3 months reported for the untreated PERMEATE cohort and markedly surpass the 5.0 months observed after multiple lines of therapy [[13], [17]]. These differences most plausibly reflect complementary, and possibly synergistic, effects of combining cranial radiotherapy with pyrotinib: radiotherapy achieves rapid intracranial cytoreduction and may disrupt the blood–brain barrier, thereby enhancing intracranial drug exposure [15], whereas pyrotinib provides sustained systemic and intracranial anti-HER2 activity. Collectively, these data support combining cranial radiotherapy with pyrotinib-based therapy to maximize intracranial control and survival in patients with HER2-positive BM.

Several considerations temper the interpretation of the null comparison across treatment sequences. First, Group C comprised only 22 patients, and the study was not powered to detect modest differences in sequence. Second, sequence was not randomly allocated; it reflected physician and patient preference as well as the clinical course, so residual selection bias is likely. Third, multivariable analysis identified age and radiotherapy modality—rather than sequence—as the dominant prognostic determinants, and these baseline and treatment factors may have overshadowed any effect of timing. Finally, Group C is intrinsically susceptible to guarantee-time bias, because assignment to this group requires survival to intracranial progression before pyrotinib can be initiated. The numerically longer OS in Group C (42.0 vs. 33.0 and 35.4 months in Groups A and B) should therefore be regarded as hypothesis- generating.

Focal radiotherapy was associated with longer OS than WBRT (adjusted HR 0.56). Sensitivity analyses showed this benefit was limited to stereotactic schedules (HR 0.52, *P* = 0.027), not moderately hypofractionated one. No radiotherapy variable predicted intracranial PFS. Focal RT was more often used for limited disease, suggesting confounding by indication, addressed by two methods. Propensity score matching reduced mean standardized differences from 0.185 to 0.100; the OS hazard ratio remained similar but non-significant. Effect direction was consistent across methods, though magnitude varied with analytic approach and eligibility. Data suggest a plausible survival advantage for focal/stereotactic RT, but not definitive superiority, supporting individualized modality choice [18]. These findings are consistent with the evolving paradigm in BM management, whereby stereotactic techniques are increasingly preferred over WBRT to achieve durable intracranial control while preserving neurocognitive function. In HER2-positive BCBM specifically, where systemic therapy with anti-HER2 agents such as pyrotinib provides meaningful intracranial activity, the value of SRS and FSRT lies in delivering high, ablative doses to discrete lesions while minimizing collateral damage to normal brain tissue—thereby complementing rather than competing with systemic treatment [13]. Two caveats deserve emphasis. First, the focal radiotherapy group in this cohort was heterogeneous, and the survival difference was concentrated in the patients treated with 3–5-fraction schedules; patients treated with moderately hypofractionated schedules had an intracranial tumour burden comparable to that of the WBRT group, and all patients with more than 10 metastases who avoided WBRT received HFRT rather than SRS or FSRT [19]. Second, neurocognitive function was not systematically assessed in this cohort, so the putative neurocognitive advantage of stereotactic techniques could not be verified here.

Multivariable analysis indicated that age > 48 years was associated with longer OS, which is contrary to the findings of previous studies [20], [21]. This association was stable across adjustment strategies—unadjusted HR 0.55; adjusted for GPA and intracranial tumour burden HR 0.54, and fully adjusted HR 0.52 —which argues against its being explained by measured baseline prognostic factors. A significant age-by-GPA interaction indicated that the survival advantage associated with older age was confined to patients with GPA 3–4 and was absent among those with GPA 0–2.5. Because the 48-year cutoff was derived from these data and the interaction was not prespecified, this observation should be regarded as exploratory. It is biologically plausible that among patients with otherwise favourable prognostic features, older age reflects less aggressive tumour biology rather than a treatment effect, whereas the detrimental influence of age is unmasked once GPA is unfavourable.

Exploratory analysis suggested that initiation of radiotherapy more than 3.9 months before pyrotinib was associated with longer OS. The direction is consistent with the numerical OS advantage seen in Group C (upfront radiotherapy: 42.0 vs. 33.0–35.4 months), but neither observation was statistically significant, and Group C is susceptible to guarantee-time bias; the two analyses therefore share a common source of bias rather than corroborating each other. If the pattern reflects a real effect, plausible mechanisms include earlier achievement of intracranial control, enhanced drug penetration across an irradiated blood–brain barrier, synergism with pyrotinib, and better preservation of neurological function, in keeping with evidence supporting early local therapy in oligometastatic disease. The cutoff was derived in 55 patients and behaved as overfitted on internal validation (permutation *P* = 0.211; optimism-corrected HR 0.66; no significance after correction for multiplicity). Our findings aligned with the numerical trend of Shumway et al. [22], but likewise did not achieve statistical significance. The 3.9-month threshold is therefore strictly hypothesis-generating and is not a clinical recommendation [23].

Whether concurrent anti-HER2 therapy increases the risk of radiation necrosis remains an important clinical concern. Radiation necrosis developed in only 7 of 101 patients (6.9%), a crude rate at the lower end of the 5–25% range reported after stereotactic irradiation of BM; the rate did not differ materially by treatment sequence or by radiotherapy modality. Small-molecule HER2 tyrosine kinase inhibitors have not shown a comparable signal elsewhere: in the phase II BROPTIMA trial, in which pyrotinib and capecitabine were administered from the first day of radiotherapy, asymptomatic necrosis was observed in 4 of 67 lesions (6.0%) treated with fractionated stereotactic radiotherapy and neurocognitive function was preserved [[7], [8], [11]]. Our findings are consistent with that pattern. Nevertheless, with only seven events and no protocol-defined neuroimaging surveillance, this study cannot exclude a modest increase in necrosis risk with concurrent pyrotinib; the question can only be resolved by prospective studies with prespecified imaging follow-up and necrosis criteria.

Finally, treatment accessibility merits comment. Pyrotinib is widely used in clinical practice across China, particularly in lower-income cities. International guidelines recommend tucatinib combined with trastuzumab and capecitabine as the preferred option for patients with active BCBM and recommend trastuzumab deruxtecan (T-DXd) as priority therapy for those with stable BM [24], [25]. In China, however, tucatinib has not yet been approved for marketing and T-DXd is not included in the National Reimbursement Drug List, which limits their accessibility. Compared with lapatinib plus capecitabine, pyrotinib plus capecitabine—which is reimbursed under the NRDL—is an alternative second-line treatment for patients with HER2-positive metastatic breast cancer in China. While T-DXd has demonstrated promising intracranial and extracranial activity with manageable adverse events, pyrotinib therefore remains a critical therapeutic option for patients with BCBM in southwestern China, a region characterized by relatively constrained economic resources.

### 4.1 Limitations

Several limitations qualify these conclusions. First, the retrospective two-centre design and the non-random allocation of both treatment sequence and radiotherapy modality leave room for residual and unmeasured confounding, which propensity-based methods may reduce but cannot eliminate. Second, detailed dosimetric data were not uniformly retrievable: whole-brain plans delivered with a simultaneous integrated or sequential boost could not be separated from whole-brain plans alone, and biologically effective dose could therefore not be analysed as a continuous variable. Third, molecular characteristics (e.g., programmed cell death ligand 1, PD-L1; tumour mutational burden, TMB) and other potent serum prognostic risk factors (such as lactate dehydrogenase, LDH) were not available for adjustment [26]. Fourth, the focal radiotherapy cohort exhibited considerable heterogeneity, with its constituent subgroups varying markedly in baseline tumour burden. Fifth, the lack of systematic assessment of neurocognitive function and health-related quality of life precluded evaluation of the hypothesized neurocognitive benefit associated with stereotactic radiotherapy. Sixth, with 70 CNS-PFS events, the study was adequately powered (80%) to detect a hazard ratio of approximately 0.51 or lower; accordingly, the non-significant findings regarding intracranial control should be regarded as inconclusive rather than as definitive evidence of equivalence. Finally, both the 3.9-month timing cutoff and the age-by-GPA interaction were post-hoc analyses and necessitate external validation in an independent cohort.

## 5 Conclusions

In this multicentre real-world cohort of 101 patients with HER2-positive breast cancer brain metastases receiving pyrotinib-based therapy, the timing of cranial radiotherapy was not significantly associated with OS or intracranial PFS, and this finding was robust to propensity score matching. Focal cranial radiotherapy were associated with improved OS. The exploratory 3.9-month radiotherapy-to-pyrotinib interval threshold is presented strictly as hypothesis-generating. Treatment-related adverse events were manageable and comparable across treatment groups. The primary conclusion is based on the direct comparison of treatment sequences; the proposed timing threshold requires prospective validation.

## Availability of data and materials

The datasets generated during and/or analysed during the current study are available from the corresponding author on reasonable request.

## Authors' contribution

All authors contributed to the study conception and design. Material preparation, data collection and analysis were performed by Dan Mao, Lan Li, Shuang Lu, Xinyan Zhou,Ye Zhang, Xuesong Chen, and Yuchao Ma, Yao-xiong Xia, Junlin Yi and Yu Hou edited and reviewed the manuscript. All authors discussed the results and provided comments on the manuscript. Zhengting Chen, Hao Jing, Sheng Huang and Miao Wang wrote the manuscript. All authors read and approved the final manuscript.

## CRediT authorship contribution statement

**Zhengting Chen:** Conceptualization, Methodology, Software, Formal analysis, Investigation, Writing – original draft, Writing – review & editing, Funding acquisition. **Hao Jing:** Conceptualization, Validation, Resources, Data curation, Writing – original draft, Funding acquisition. **Sheng Huang:** Conceptualization, Validation, Resources, Data curation, Writing – original draft, Funding acquisition. **Miao Wang:** Conceptualization, Formal analysis, Data curation, Writing – original draft. **Dan Mao:** Methodology, Software, Formal analysis, Data curation, Investigation, Visualization. **Lan Li:** Investigation, Data curation. **Shuang Lu:** Investigation, Data curation. **Xinyan Zhou:** Investigation, Data curation. **Dongyu Bi:** Investigation, Data curation. **Ye Zhang:** Investigation, Resources, Data curation. **Xuesong Chen:** Investigation, Data curation. **Yuchao Ma:** Investigation, Data curation, Validation. **Yaoxiong Xia:** Resources, Project administration, Supervision, Writing – review & editing, Funding acquisition. **Junlin Yi:** Resources, Project administration, Supervision, Writing – review & editing. **Yu Hou:** Resources, Project administration, Supervision, Writing – review & editing.

## Ethics approval and consent to participate

This is an observational study. The present study was in compliance with the Declaration of Helsinki. This retrospective study was approved by the Medical Ethics Committee of the Third Affiliated Hospital of Kunming Medical University (Approval No. KYLX2024–066). The requirement for informed consent was waived owing to the retrospective nature of the analysis.

## Funding

This work was supported by the National Natural Scientific Foundation of China (Grant No. 82560568,82403353), supported by the Applied Basic Research in Yunnan Province (Joint Special Project of Kunming Medical University) (Grant No. 202401AY070001–151, 202601AY070001–016), supported by the Beijing Hope Run Special Fund of Cancer Foundation of China (Grant No. LC2024A15), supported by the Chronic Disease Management Research Project of National Health Commission Capacity Building and Continuing Education Center (Grant No. GWJJMB202510010003), supported by the Yunnan Province Clinical Medicine Center Research Project (Grant No. 2024YNLCYXZX0403).

## Declaration of competing interest

The authors declare that they have no known competing financial interests or personal relationships that could have appeared to influence the work reported in this paper.
