# Supplementary material for: Timing of cranial radiotherapy in HER2-positive breast Cancer brain metastases treated with Pyrotinib: a multicentre real-world cohort study
Source: Clin Transl Radiat Oncol. 2026 Oct 7;62:101310. doi: 10.1016/j.ctro.2026.101310 (PMC13695302; doi:10.1016/j.ctro.2026.101310)
Supplement: Supplementary file 1 [file mmc1.docx]

Figure S1. Patient flowchart.

Table S1. Clinical circumstances in which cranial radiotherapy was delivered, according to treatment sequence (N = 101)

| **Circumstance** | **Group A (n = 41)** | **Group B (n = 38)** | **Group C (n = 22)** | **P value** |
| --- | --- | --- | --- | --- |
| Principal indication for the timing of radiotherapy |  |  |  |  |
| Symptomatic brain metastases at the time radiotherapy was scheduled | 24 (58.5) | 21 (55.3) | 11 (50.0) | 0.81 |
| Asymptomatic brain metastases detected on staging or surveillance imaging | 17 (41.5) | 17 (44.7) | 11 (50.0) | 0.81 |
| Intracranial progression during pyrotinib-based therapy as the trigger for radiotherapy | 0 | 38 (100) | 0 | — |
| Intracranial progression after radiotherapy as the trigger for pyrotinib | 0 | 0 | 22 (100) | — |
| No progression trigger; both modalities administered concurrently | 41 (100) | 0 | 0 | — |
| Intracranial disease burden at the time of radiotherapy |  |  |  |  |
| ≤4 brain metastases | 28 (68.3) | 22 (57.9) | 15 (68.2) | 0.57 |
| >4 brain metastases | 13 (31.7) | 16 (42.1) | 7 (31.8) | 0.57 |
| >10 brain metastases | 1 (2.4) | 5 (13.2) | 3 (13.6) | 0.38 |
| Maximum lesion diameter ≥3 cm | 8 (19.5) | 6 (15.8) | 3 (13.6) | 0.82 |
| Systemic disease status |  |  |  |  |
| Extracranial metastases present (at any time) | 23 (56.1) | 27 (71.1) | 17 (77.3) | 0.18 |
| ≥3 prior lines of systemic therapy | 10 (24.4) | 19 (50.0) | 7 (31.8) | 0.046 |
| Guarantee (immortal) time |  |  |  |  |
| Median interval from brain metastasis diagnosis to the second modality, months | 1.1 | 7.7 | 18.4 | — |
| As a proportion of median overall survival, % | 5.6 | 33.4 | 48.1 | — |

Values are n (%) unless otherwise stated. Groups: A, concurrent pyrotinib and radiotherapy; B, radiotherapy after intracranial progression on pyrotinib; C, pyrotinib after progression following radiotherapy. P values are from chi-square tests across the three groups; "—" denotes categories defined by group assignment and therefore not tested. Abbreviations: BM, brain metastases; NA, not available; RT, radiotherapy.

Table S2. Radiotherapy modality, dose–fractionation schedules and biologically effective dose (N = 101)

| Modality | Patients, n (%) | Dose–fractionation schedules | BED10 (Gy)  median (range) | EQD2 (Gy)  median (range) |
| --- | --- | --- | --- | --- |
| WBRT ± boost | 43 (42.6) | 30 Gy / 10 F; 40 Gy / 20 F;  SIB 49.5–60 Gy/10–20 F | 39.0; 48.0;  70.9(65.8-81.3) | 32.5; 40.0;  59.1 (54.9-67.8) |
| SRT | 34 (33.7) | 24–35 Gy in 3–5 F | 48.0 (43.2–60.0)‡ | 44.8(36.0–50.0) |
| HFRT | 22 (21.8) | 36–58.5 Gy in 12–20 F | 70.88 (46.8–74.8) | 59.06 (39.0–62.4) |
| Other / NR | 2 (2.0) | — | — | — |

BED10 = D × (1 + d / 10), assuming α/β = 10 Gy for tumour; EQD2 = BED10 / 1.2. D, total dose; d, dose per fraction. ‡Range covering the schedules (24 Gy/3 F, 30 Gy/5 F, 35 Gy/5 F, 30 Gy/3 F). Abbreviations: BED, biologically effective dose; EQD2, equivalent dose in 2-Gy fractions; NR, not retrievable; SRT, stereotactic radiotherapy; HFRT, hypofractionated radiotherapy; SIB, simultaneous integrated boost; WBRT, whole-brain radiotherapy.

**Propensity score matching — focal radiotherapy versus WBRT**

In the propensity score–matched cohort (24 pairs; 48 patients), the median OS was 40.3 months with focal radiotherapy and 28.0 months with WBRT (log-rank P = 0.086). The hazard ratio for death was 0.54 (95% CI 0.29–1.01; P = 0.052) using a cluster-robust variance estimator and 0.64 (95% CI 0.25–1.64; P = 0.350) in a model stratified on the matched pair. The point estimate was essentially unchanged from the unmatched analysis, while the confidence interval widened appreciably owing to the reduction in sample size. Covariate balance improved (mean |SMD| 0.185 to 0.100) but was not complete; age, GPA and lesion number remained slightly imbalanced. An IPTW analysis retaining all 91 patients achieved better balance (mean |SMD| 0.066) and yielded an HR of 0.52 (95% CI 0.31–0.88; P = 0.015) for OS, consistent with the primary multivariable analysis(Table S3, Figure S2).

Table S 3.1-PSM1: Baseline Characteristics and Standardized Mean Differences Before and After Matching

| Covariate | Focal RT (n = 52) | WBRT (n = 39) | SMD before | Focal RT (n = 24) | WBRT (n = 24) | SMD after |
| --- | --- | --- | --- | --- | --- | --- |
| Age >48 y | 30 (57.7) | 15 (38.5) | 0.388 | 8 (33.3) | 11 (45.8) | -0.252 |
| KPS score | 84.9 (9.4) | 84.5 (9.4) | 0.044 | 84.8 (10.5) | 85.2 (9.5) | -0.042 |
| GPA 3-4 | 27 (51.9) | 25 (64.1) | -0.246 | 17 (70.8) | 15 (62.5) | 0.174 |
| BM number >4 | 15 (28.8) | 20 (51.3) | -0.465 | 8 (33.3) | 10 (41.7) | -0.169 |
| BM number >10 | 6 (11.5) | 3 (7.7) | 0.129 | 1 (4.2) | 1 (4.2) | 0.000 |
| BM size >=3 cm | 10 (19.2) | 5 (12.8) | 0.173 | 5 (20.8) | 4 (16.7) | 0.105 |
| BM symptomatic | 30 (57.7) | 22 (56.4) | 0.026 | 13 (54.2) | 14 (58.3) | -0.082 |
| Extracranial metastases | 31 (59.6) | 29 (74.4) | -0.314 | 15 (62.5) | 16 (66.7) | -0.085 |
| Prior therapy >=3 lines | 18 (34.6) | 16 (41.0) | -0.131 | 7 (29.2) | 8 (33.3) | -0.088 |
| Sequence: B vs A | 21 (40.4) | 15 (38.5) | 0.039 | 9 (37.5) | 9 (37.5) | 0.000 |
| Sequence: C vs A | 9 (17.3) | 8 (20.5) | -0.081 | 4 (16.7) | 5 (20.8) | -0.105 |
| Mean \|SMD\| |  |  | 0.185 |  |  | 0.100 |
| Max \|SMD\| |  |  | 0.465 |  |  | 0.252 |

Values are n (%) or mean (SD). SMD, standardized mean difference. Focal radiotherapy = SRT/mHFRT. Analysed cohort n = 91 (KPS complete cases); 28 focal and 15 WBRT patients were unmatched and excluded. PS range: focal 0.167–0.982, WBRT 0.079–0.920.

Table S3.2 PSM2: Post-matching Cox Regression

| Endpoint | Analysis | n | HR (95% CI) | P value |
| --- | --- | --- | --- | --- |
| OS | Unmatched (analysis cohort, n = 91) | 91 | 0.54 (0.32–0.91) | 0.021 |
| **OS** | **Matched, cluster-robust SE (24 pairs)** | **48** | **0.54 (0.29–1.01)** | **0.052** |
| OS | Matched, stratified on matched pair | 48 | 0.64 (0.25–1.64) | 0.350 |
| OS | Matched, naive Cox (no clustering) | 48 | 0.54 (0.26–1.10) | 0.091 |
| CNS-PFS | Unmatched (analysis cohort, n = 91) | 91 | 0.71 (0.43–1.17) | 0.182 |
| CNS-PFS | Matched, cluster-robust SE (24 pairs) | 48 | 0.63 (0.35–1.15) | 0.134 |
| CNS-PFS | Matched, stratified on matched pair | 48 | 0.45 (0.16–1.31) | 0.144 |

HR < 1 favours focal radiotherapy. Cluster-robust sandwich variance with the matched pair as the clustering unit. In the matched cohort: OS events 12 (focal) vs 21 (WBRT), median OS 40.3 vs 28.0 months, log-rank *P* = 0.086; CNS-PFS events 13 vs 22, median 24.0 vs 11.8 months, log-rank *P* = 0.195.

Table S3.3 Sensitivity Analysis

| Analysis | n | Pairs | Mean \|SMD\| after | OS HR (95% CI) | OS P | CNS-PFS HR (95% CI) |
| --- | --- | --- | --- | --- | --- | --- |
| P1 Primary: 1:1 NN, caliper 0.2 SD (complete cases) | 91 | 24 | 0.100 | 0.54 (0.29–1.01) | 0.052 | 0.63 (0.35–1.15) |
| S1 Caliper 0.1 SD | 91 | 19 | 0.127 | 0.64 (0.29–1.41) | 0.271 | 0.76 (0.40–1.45) |
| S2 Caliper 0.5 SD | 91 | 27 | 0.100 | 0.60 (0.31–1.17) | 0.137 | 0.73 (0.40–1.34) |
| S3 Optimal (Hungarian) matching, 0.2 SD | 91 | 24 | 0.183 | 0.61 (0.32–1.18) | 0.143 | 0.66 (0.36–1.23) |
| S4 KPS missing imputed as 90 (n = 101) | 101 | 26 | 0.142 | 0.69 (0.37–1.30) | 0.256 | 0.79 (0.40–1.56) |
| S5 SRS/FSRT (n = 30) vs WBRT | 69 | 14 | 0.222 | 1.02 (0.44–2.39) | 0.955 | 0.77 (0.33–1.80) |
| S6 IPTW (ATT, stabilized, trimmed) | 91 | — | 0.066 | 0.52 (0.31–0.88) | 0.015 | 0.62 (0.37–1.04) |

S1–S5 HRs from cluster-robust Cox on the matched set; S6 from IPTW-weighted Cox with robust SE. IPTW: stabilized ATT weights 0.48–3.61, effective sample size 64.9, mean |SMD| 0.185 → 0.066, max |SMD| 0.465 → 0.164. S5 (SRS/FSRT vs WBRT) yielded only 14 pairs with residual imbalance (max |SMD| 0.575) and should be regarded as exploratory.

Figure S2. Covariate balance before and after 1:1 propensity score matching (love plot). Grey band denotes |SMD| < 0.1.

Fig. S3.1. Restricted cubic spline of the association between radiotherapy timing and overall survival.

Fig. S3.2. Instability of the data-derived cutoff for the radiotherapy-to-pyrotinib interval.
